# Structure from Articulated Motion: Accurate and Stable Monocular 3D Reconstruction without Training Data

**DOI:** 10.3390/s19204603

**Published:** 2019-10-22

**Authors:** Onorina Kovalenko, Vladislav Golyanik, Jameel Malik, Ahmed Elhayek, Didier Stricker

**Affiliations:** 1 Department Augmented Vision, German Research Center for Artificial Intelligence (DFKI), 67663 Kaiserslautern, Germany; jameel.malik@dfki.de (J.M.); ahmed.elhayek@dfki.de (A.E.); didier.stricker@dfki.de (D.S.); 2 Department of Computer Graphics, Max Planck Institute for Informatics, 66123 Saarbrücken, Germany; golyanik@mpi-inf.mpg.de; 3 Department of Computer Science, University of Kaiserslautern, 67663 Kaiserslautern, Germany; 4 School of Electrical Engineering and Computer Science (SEECS), National University of Sciences and Technology (NUST), 44000 Islamabad, Pakistan; 5 Department of Computer Science, University of Prince Mugrin (UPM), 20012 Madinah, Saudi Arabia

**Keywords:** structure from motion, human pose estimation, articulated structure recovery

## Abstract

Recovery of articulated 3D structure from 2D observations is a challenging computer vision problem with many applications. Current learning-based approaches achieve state-of-the-art accuracy on public benchmarks but are restricted to specific types of objects and motions covered by the training datasets. Model-based approaches do not rely on training data but show lower accuracy on these datasets. In this paper, we introduce a model-based method called *Structure from Articulated Motion* (SfAM), which can recover multiple object and motion types without training on extensive data collections. At the same time, it performs on par with learning-based state-of-the-art approaches on public benchmarks and outperforms previous non-rigid structure from motion (NRSfM) methods. SfAM is built upon a general-purpose NRSfM technique while integrating a soft spatio-temporal constraint on the bone lengths. We use alternating optimization strategy to recover optimal geometry (i.e., bone proportions) together with 3D joint positions by enforcing the bone lengths consistency over a series of frames. SfAM is highly robust to noisy 2D annotations, generalizes to arbitrary objects and does not rely on training data, which is shown in extensive experiments on public benchmarks and real video sequences. We believe that it brings a new perspective on the domain of monocular 3D recovery of articulated structures, including human motion capture.

## 1. Introduction

3D structure recovery of articulated objects (i.e., comprising multiple connected rigid parts) from a set of 2D point tracks through multiple monocular images is a challenging computer vision problem [1,2,3,4]. Articulated structure recovery is ill-posed due to missing information about the third dimension [5]. Its applications include gesture and activity recognition, character animation in movies and games, and motion analysis in sport and robotics.

Recently, multiple learning-based approaches that recover 3D structures from 2D landmarks have been introduced [6,7,8,9]. These methods show state-of-the-art accuracy across public benchmarks. However, they are restricted to a specific kind of structure (e.g., human skeleton) and require extensive datasets for training. Moreover, they often fail to recover poses that are different from the training examples (see Section 4.2.5). When a scene includes different types of articulated objects, different methods have to be applied to reconstruct the whole scene.

**Figure 1:**
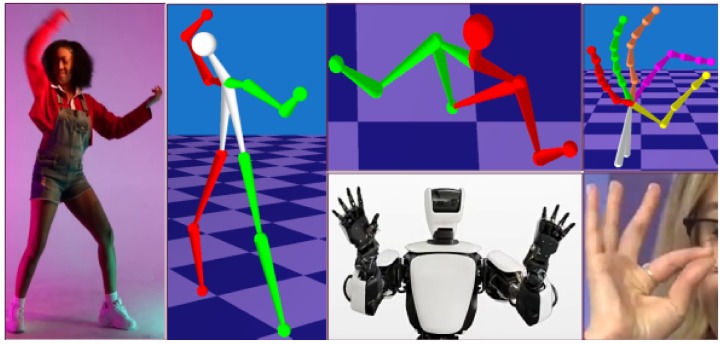
We recover different articulated structures from real-world videos with high accuracy and no need for training data. Our *Structure from Articulated Motion* (SfAM) approach is not restricted to a single object class and only requires a rough articulated structure prior. The reconstructions are provided under different view angles.

In this paper, we introduce a general approach for accurate recovery of 3D poses of any articulated structure from 2D observations that does not rely on training data (see Figure 1). We build upon the recent progress in non-rigid structure from motion (NRSfM), which is a general technique for non-rigid 3D reconstruction from 2D point tracks. However, when considering an articulated object as a general non-rigid one, reconstructions can evince significant variations in the distances between the connected joints (see Section 4.2.3). These distances have to remain nearly constant across all articulated poses. Our method relies on this assumption and imposes a spatio-temporal constraint on the bone lengths.

**Figure 2:**
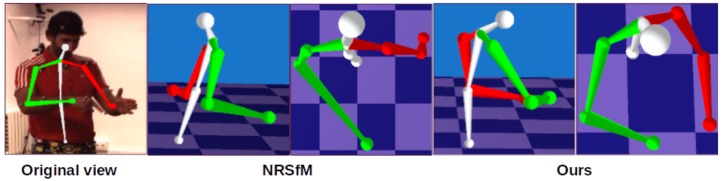
Side-by-side comparison of the non-rigid structure from motion (NRSfM) method [11] and our SfAM. Reconstruction results of [11] violate anthropometric properties of the human skeleton due to changing bone lengths from frame to frame.

We call our approach *Structure from Articulated Motion* (SfAM). We apply an articulated structure term as a soft constraint on top of the classic optimization problem of NRSfM [10]. This term enforces the bone lengths—though not known in advance—to remain constant across all frames. Our optimization strategy alternates between the classic NRSfM problem and our articulated structure term until they both converge. This allows for recovering the geometry together with the 3D joint positions and the method does not rely on known bone lengths. Starting from a rough initialization of the articulated structure (e.g., a human arm is longer than a leg), SfAM still converges to the correct structure proportions (see Section 4.2.3). Figure 2 illustrates the significant difference between results produced by a general-purpose NRSfM technique [11] and our SfAM.

To summarise, our **contributions** are:

- **A generic framework** for articulated structure recovery which achieves state-of-the-art accuracy among not learning-based methods across public datasets. Moreover, it shows performance close to state-of-the-art learning-based methods but at the same time is not restricted to specific objects (see Section 4) and does not require training data.
- **SfAM recovers sequence-specific bone proportions** together with 3D joints (see Section 3). Thus, it does need known bone lengths.
- The **articulated prior energy term** makes our approach robust to noisy 2D observations (see Section 4.2.2) by imposing additional constraints on the 3D structure.

In this paper, we show that a not learning-based approach can perform on par with state-of-the-art learning-based methods and even outperform some of them in real-world scenes (see Section 4.2.5). We demonstrate the effectiveness of SfAM for the recovery of different articulated structures through extensive quantitative and qualitative evaluation on different datasets [12,13,14] and real-world scenes (see Section 4). To the best of our knowledge, our SfAM is the first NRSfM approach evaluated on such comprehensive datasets as Human 3.6m [12] and NYU hand pose [14]. As a side effect of our method, it can be used for precise articulated model estimation (generate personalized human skeleton rigs (see Section 4.2.3)). This contrasts a lot with most recent supervised learning approaches which require extensive labeled databases for training, and still, often fail when unfamiliar poses are observed (see Section 4.2.5). Moreover, minor changes in the inputs lead to significant variations in the poses, which makes the results of learning-based methods very difficult or impossible to reproduce.

## 2. Related Work

**Rigid and Non-Rigid Structure from Motion.** Factorization-based Structure from Motion (SfM) is a general technique for 3D structure recovery from 2D point tracks. An SfM problem is well-posed for rigid objects due to the rigidity constraint [15]. Early extensions of Tomasi and Kanade’s method [15] for the non-rigid case rely on rank and orthonormality constraints [16,17]. Subsequent methods investigated shape basis priors [18], temporal smoothness priors [19], trajectory space constraints [20] as well as such fundamental questions as shape basis uniqueness [21,22]. More recent methods combine priors in the metric and trajectory spaces  [23]. To improve the reconstruction of stronger nonlinear deformations, Zhu et al. [24] introduce unions of linear subspaces. Dai et al. [10] propose an NRSfM method with as few additional constraints as possible. Lately, the focus of NRSfM research is drawn to the problem of scalability [11,25], i.e., the consistent performance across different scenarios and linear computational complexity in the number of points. Our SfAM is a scalable approach which builds upon the work of Ansari et al. [11]. In contrast to [11], we recover articulated structures with higher accuracy.

**Articulated and Multibody Structure from Motion.** Over the last few years, several SfM approaches for articulated motion recovery were proposed. Some of them relax the global rigidity constraint for multiple parts [26,27] so that each of the parts is constrained to be rigid. They can handle relatively simple articulated motions, as the segmentation and the structure composition are assumed to be unknown [26]. As a result, these methods are hardly applicable to such complicated scenarios as human and hand pose recovery. Tresadern and Reid [28], Yan and Pollefeys [29] and Palladini et al. [26] address the articulated case with two rigid body parts and detect a hinge joint. Later, an approach with spatial smoothness and segmentation dealing with an arbitrary number of rigid parts was proposed by Fayad et al. [30]. Park and Sheikh [31] reconstruct trajectories given parent trajectories and known bone length, known camera, and root motion for each frame. Their objective is highly nonlinear and requires good initialization of trajectory parameters. In contrast, our method recovers sequence-specific bone proportions and does not rely on given bone lengths. Next, Valmadre et al. [32] propose a dynamic-programming approach for the reconstruction of articulated 3D trees from input 2D joint positions operating in linear time. Multibody SfM methods reconstruct multiple independent rigid body transformations and non-rigid deformations in the same scene [27,33]. In contrast, our approach is more general as it imposes a soft constraint of articulated motion on top of classic NRSfM.

**Piecewise and Locally Rigid Structure from Motion.** Piecewise rigid approaches interpret the structure as locally rigid in the spatial domain [34,35]. Several methods divide the structure into patches, each of which can deform non-rigidly [36,37]. High granularity level of operation allows these methods to reconstruct large deformations as opposed to methods relying on linear low-rank subspace models [36]. Rehan et al. [38] penalize deviations between the bone lengths from the average distances between the joints over the whole sequence. This form of constraint does not guarantee a realistic reconstruction though, as it struggles to compensate for inaccurate 2D estimations or 3D inaccuracies in short time intervals.

**Monocular 3D Human Body and Hand Pose Estimation.** Bone length constraints are widely used in the single-view regression of 3D human poses. One of the early works in this domain operates on single uncalibrated images and imposes constraints on the relative bone lengths [39]. It is capable of reconstructing a human pose up to scale. Later, an enhancement for multiple frames with bone symmetry and rigidity constraints (joints representing the same bone move rigidly relative to each other) was introduced by Wei and Chai [40]. Akhter and Black [41] use a pose prior that captures pose-dependent joint angle limits. Ramakrishna et al. [1] use a sum of squared bone lengths term that can still lead to unrealistic poses. Wandt et al. [2] constrain the bone lengths to be invariant. Their trilinear factorization approach relies on pre-trained body poses serving as a shape prior and transcendental functions modeling periodic motion peculiar to the human gait. An adaptation of this approach to hand gestures would require the acquisition of a new shape prior. Wandt et al. [42] constrain the sum of squared bone lengths of the articulated structure to be invariant throughout image sequence. However, the length of each bone can still vary. One of the modern methods for human pose and appearance estimation is MonoPerfCap of Xu et al. [43]. It imposes implicit bone length constraints through a dense template tailored to a specific person and captured in an external acquisition process.

Recently, many learning-based approaches for human pose and hand pose estimation have been presented in the literature  [9,44,45,46,47,48,49,50,51]. In [7], weak supervision constrains the output of the network with fixed bone proportions taken from the training dataset. Sun et al. [52] exploit a joint connection structure and uses bones instead of joints for pose representation. Wandt and Rosenhahn [53] use kinematic chain representation and include bone length information to their loss function during training. In contrast to our SfAM, [53] is not as robust to noisy 2D input (see Section 4.2.2). All these methods are highly specialized and rely on extensive collections of training data. In contrast, our SfAM is a general approach that can cope with different articulated structures, with no need for labeled datasets.

## 3. The Proposed SfAM Approach

**Figure 3:**
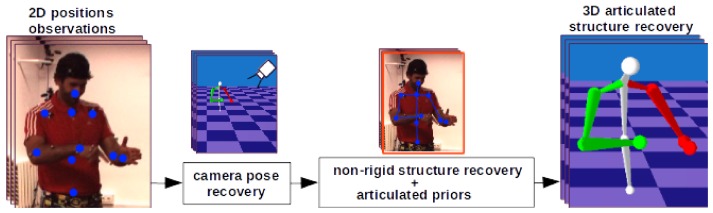
The pipeline of the proposed SfAM approach. Following factorization-based NRSfM, we first recover the camera pose using 2D position observations. Then, we recover 3D articulated structure by optimizing our new energy functional accounting for articulated priors.

Figure 3 shows a high-level overview of our approach. Following factorization-based NRSfM [10], we first recover the camera pose using 2D landmarks (Section 3.2). For 3D structure recovery, we extend the target energy function of the classic NRSfM problem [10,11] by our articulated prior term (Section 3.3.1).

We assume that sparse 2D correspondences are given. In Section 3.3.2, we show how our new energy is efficiently optimized alternating between fixed-point continuation algorithm [54] and Levenberg–Marquardt [55,56]. This leads to an accurate reconstruction of articulated motions of different structures.

### 3.1. Factorization Model

The input to SfAM is the measurement matrix $W={\left[W_{1},W_{2},\cdots ,W_{T}\right]}^{T}\in R^{2T\times N}$ with *N* 2D joints tracked over *T* frames. Every $W_{t}$, t∈{1,⋯,T}, is registered to the centroid of the observed structure and the translation is resolved in advance. Most of the NRSfM methods assume orthographic projection, as the intrinsic camera model is usually not known. Even though some benchmarks (e.g., [12]) provide camera parameters, we develop a general approach for uncalibrated settings. Following standard SfM approaches, we assume that every 2D projection $W_{t}$ can be factorized into a camera pose-projection matrix $R_{t}\in R^{2\times 3}$ and 3D structure $S_{t}\in R^{3\times N}$ so that $W_{t}=R_{t}S_{t}$. We assume that the articulated structure deforms under the low-rank shape model [11,16]. Thus, $S={\left[S_{1},S_{2},\cdots ,S_{T}\right]}^{T}$ can be parametrized by the set of unknown basis shapes $B\in R^{3K\times N}$ of cardinality *K* and the coefficient matrix $C\in R^{T\times K}$:

(1) $$\begin{array}{c} W=RS=\underset{M}{\underset{︸}{R\;(C⊗I_{3})}}B=MB, \end{array}$$

where $R=\mathrm{bkdiag}(R_{1},R_{2},\ldots ,R_{T})$ is the joint camera pose-projection matrix, $I_{3}$ is a 3×3 identity matrix and ⊗ denotes Kronecker product.

### 3.2. Recovery of Camera Poses

Applying singular value decomposition to W, we obtain initial estimates of M and B from Equation (1) up to an invertible corrective transformation $Q\in R^{3K\times 3K}$:

(2) $$\begin{array}{c} W≅M^{\prime }B^{\prime }≅\underset{M}{\underset{︸}{M^{\prime }Q}}\;\underset{B}{\underset{︸}{Q^{-1}B^{\prime }}}=MB. \end{array}$$

In the following, we are using the shortcuts $M_{2t-1:2t}^{\prime }\in R^{2\times 3K}$ for every *t*-th pair of rows of M, $Q_{k}\in R^{3K\times 3}$ for the *k*-th column triplet of Q, k∈{1,…,K}. Considering (1) and (2), for every t∈{1,…,T} and k∈{1,…,K}, we have:

(3) $$\begin{array}{c} M_{2t-1:2t}^{\prime }Q_{k}=c_{tk}R_{t}. \end{array}$$

Using the orthonormality constraints $R_{t}R_{t}^{T}=I_{2}$ and denoting $F=QQ^{T}$, we obtain:

(4) $$\left\{\begin{array}{c} M_{2t-1}^{\prime }F_{k}M_{2t-1}^{\prime T}=M_{2t}^{\prime }F_{k}M_{2t}^{\prime T}=c_{ik}^{2}I_{2},\\ M_{2t-1}^{\prime }F_{k}M_{2t}^{\prime T}=0. \end{array}\right.$$

Therefore, the following systems of equations can be written for every *t* and *k*:

(5) $$\begin{array}{c} \underset{G_{t}}{\underset{︸}{\left[\begin{array}{c} M_{2t-1}^{\prime }⊗M_{2t-1}^{\prime T}-M_{2t}^{\prime }⊗M_{2t}^{\prime T}\\ M_{2t-1}^{\prime }⊗M_{2t}^{\prime T} \end{array}\right]}}\mathrm{vec}\left(F_{k}\right)=0, \end{array}$$

where vec(·) is vectorization operator permuting a m×n matrix to a mn column vector. Stacking all $G_{t}$ vertically, we obtain:

(6) $$\begin{array}{c} G\mathrm{vec}(F_{k})=0, \end{array}$$

where $G={\left[G_{1},G_{2},\ldots ,G_{T}\right]}^{T}$. Finding an optimal $F_{k}$ can be performed by solving the optimization problem:

(7) $$\begin{array}{c} \min_{\;\;F_{k}}{∥G\mathrm{vec}(F_{k})∥}^{2}. \end{array}$$

Due to the rank-3 constraint on every $F_{k}$, this problem is solved by the iterative shrinkage-thresholding (IST) method [57]. Once an optimal F is found, the corrective transformation Q is recovered by Cholesky decomposition. Using Q, R is recovered from Equations (1)–(4).

### 3.3. Articulated Structure Recovery

#### 3.3.1. Articulated Structure Representation

Having found R, we recover S. Note that we optionally rely on an updated W after the smooth shape trajectory step which imposes additional constraints on point trajectories and reduces the overall number of unknowns; please refer to [11] for more details. We rearrange the shape matrix S to

(8) $$S^{\#}=\left[\begin{array}{ccc} X_{11}\ldots X_{1N} & Y_{11}\ldots Y_{1N} & Z_{11}\ldots Z_{1N}\\ \vdots \;\;\;\vdots & \vdots \;\;\;\vdots & \vdots \;\;\;\vdots \\ X_{T1}\ldots X_{TN} & Y_{T1}\ldots Y_{TN} & Z_{T1}\ldots Z_{TN} \end{array}\right],$$

where $\left(X_{tn},Y_{tn},Z_{tn}\right),n\in \left\{1,\ldots ,N\right\}$ is a 3D coordinate of each joint in S. $S^{\#}$ can be represented as:

(9) $$S^{\#}=\left[P_{x}P_{y}P_{z}\right]\left(I_{3}⊗S\right),$$

where $P_{x},P_{y},P_{z}\in R^{T\times 3N}$ are binary row selectors. We follow [10,11] and represent the optimal non-rigid structure by:

(10) $$\min_{S}\left|\right|S^{\#}\Pi {\left|\right|}_{*},\;s.\;t.\;W=RS,$$

where $\Pi =(I-\frac{1}{T}11^{T})$ (1 is a vector of ones) and ${\left||.|\right|}_{*}$ denotes the nuclear norm. Note that $\mathrm{rank}(S^{\#})\leq K$, and the mean 3D component is removed from $S^{\#}$. As shown in Figure 2, non-rigid structures recovered by the optimization of (10) can have significant variations in bone lengths. This often leads to unrealistic poses and body proportions. Unlike general non-rigid structures, in articulated structures, individual rigid parts or bones have constant lengths throughout the whole sequence. Moreover, all the bones follow constant proportions. These constraints are called *articulated priors*. We incorporate the articulated priors into the objective function (10) in the form of the following energy term:

(11) $$E_{BL}\left(S\right)=\sum_{t=1}^{T}\sum_{b=1}^{B}e_{tb}\left(S\right),$$

where $e_{tb}\left(S\right)={\left(D_{b}^{t}-L_{b}\right)}^{2}$ is an energy term for bone *b* and frame *t*, $L_{b}$ is initial normalized bone length value of bone *b*. The normalization is done with respect to the sum of all initial bone lengths. $D_{b}^{t}=\left|\right|X_{a_{b}}^{t}-X_{c_{b}}^{t}{\left|\right|}_{2}$ is Euclidian distance between joints $X_{a_{b}}^{t}$ and $X_{c_{b}}^{t}$ connected by bone *b*; *B* is the number of bones of the articulated structure. Vectors $a=[X_{a_{1}},X_{a_{2}},\ldots ,X_{a_{B}}]$ and $c=[X_{c_{1}},X_{c_{2}},\ldots ,X_{c_{B}}]$ define the parent and child joints of bones, respectively.

Unlike some previous works [7,41,58,59], we do not require predefined bone lengths or proportions. SfAM recovers optimal articulated structure that minimizes the total energy:

(12) $$\min_{S}\left(\left|\right|S^{\#}{\left|\right|}_{*}+\frac{\beta }{2}E_{BL}\left(S\right)\right),\;s.\;t.\;W=RS,$$

where β is a scalar weight. Implementation of articulated prior (11) as a soft constraint makes the overall method robust to incorrect initialization of bone lengths.

#### 3.3.2. Energy Optimization

Since (12) contains a nonlinear term $E_{BL}\left(S\right)$, we introduce an auxiliary variable A and obtain the following optimization problem which is linear with respect to S:

(13) $$\begin{array}{cc} \; & \min_{S}\left|\right|S^{\#}{\left|\right|}_{*}+\frac{\beta }{2}\min_{A}E_{BL}\left(A\right),\\ \; & \;\;s.\;t.\;\;W=RS\;\mathrm{and}\;A=S. \end{array}$$

We rewrite (13) in the Lagrangian form:

(14) $$\begin{array}{cc} \; & L\left(S,A,\mu \right)=\mu ||S^{\#}{\left|\right|}_{*}+\frac{\beta }{2}E_{BL}\left(A\right)+\frac{1}{2}||W-{RS||}_{F}^{2}+\frac{1}{2}||A-{S||}_{F}^{2}, \end{array}$$

where ${\left||.|\right|}_{F}$ denotes the Frobenius norm and μ is a parameter. We split 14 into two subproblems:

(15) $$\begin{array}{ccc} \; & \min_{S}L\left(S,\mu \right)= & \min_{S}\left(\mu ||S^{\#}{\left|\right|}_{*}+\frac{1}{2}||W-{RS||}_{F}^{2}+\frac{1}{2}||A-{S||}_{F}^{2}\right) \end{array}$$

(16) $$\begin{array}{cc} \; & \;\mathrm{and}\;\min_{A}L\left(A\right)=\min_{A}\left(\frac{\beta }{2}E_{BL}\left(A\right)+\frac{1}{2}||A-{S||}_{F}^{2}\right). \end{array}$$

We alternate between the subproblems (15) and (16) and iterate until convergence. A remains fixed in (15) and S remains fixed in (16). In every optimization step, the subproblem (15) updates the 3D structure so that it more accurately projects to the observed 2D landmarks. The subproblem (16) penalizes the difference in bone lengths among all frames while recovering the sequence-specific bone proportions. The bone lengths of the recovered optimal 3D structures are almost constant throughout the whole image sequence but different from the initial $L_{b}$.

The subproblem (15) is linear and solved by the fixed-point continuation (FPC) method [54]. First, we obtain the gradient of $\frac{1}{2}(||W-{RS||}_{F}^{2}+||A-{S||}_{F}^{2})$ with respect to $S^{\#}$:

(17) $$\begin{array}{c} g\left(S^{\#},A\right)=\frac{\partial \frac{1}{2}(||W-{RS||}_{F}^{2}+||A-{S||}_{F}^{2})}{\partial S^{\#}}=\left[P_{x}P_{y}P_{z}\right]\left(I_{3}⊗\left(R^{T}\left(RS-W\right)+\left(S-A\right)\right)\right). \end{array}$$

Next, FPC for $\min_{S}L\left(S,\mu \right)$ instantiates as:

(18) $$\begin{array}{cc} \; & Y^{\left(t+1\right)}=S^{\#(t)}-\tau g\left(S^{\#(t)},A^{\left(t\right)}\right),\\ \; & \;S^{\#(t+1)}=S_{\tau \mu^{\left(t\right)}}\left(Y^{\left(t+1\right)}\right),\\ \; & \;\mu^{\left(t+1\right)}=\rho \mu^{\left(t\right)}, \end{array}$$

where $S_{\nu }\left(\cdot \right)$ is the matrix shrinkage operator [54] and τ>0 is a free parameter.

The second subproblem (16) is nonlinear and is optimized for each iteration (18) using Levenberg–Marquardt of *ceres*  [60]. Let denote the $r_{l}$, l∈{1,…,TN} residuals of $\frac{1}{2}||A-{S||}_{F}^{2}$. We aggregate all residuals $e_{tb}\left(A\right)$ from (11) (note that S in (11) is substituted by A) and $r_{l}$ into a single function:

(19) $$\begin{array}{cc} F(A)= & {\left[e_{11}\left(A\right),\ldots ,e_{BT}\left(A\right),r_{1},\ldots ,r_{TN}\right]}^{T}:\\ & R^{3TN}\to R^{BT+TN}. \end{array}$$

Next, the objective function (16) can be compactly written in terms of A as:

(20) $$\begin{array}{c} L\left(A\right)={∥F(A)∥}_{2}^{2}. \end{array}$$

The target nonlinear energy optimization problem consists of finding an optimal parameter set $A^{\prime }$ so that:

(21) $$\begin{array}{c} A^{\prime }=\arg\min_{A}{∥F(A)∥}_{2}^{2}. \end{array}$$

We solve (21) iteratively. In every optimization step *k*, the objective is linearized in the vicinity of the current solution $A_{k}$ by the first-order Taylor expansion:

(22) $$\begin{array}{c} F\left(A_{k}+\Delta A\right)\approx F\left(A_{k}\right)+J\left(A_{k}\right)\Delta A, \end{array}$$

with $J{\left(A\right)}_{(BT+TN)\times 3TN}$ being the Jacobian of $F(A_{k})$. For every iteration, the objective for ΔA reads:

(23) $$\begin{array}{c} \min_{\Delta A}{∥J\left(A_{k}\right)\Delta A+F\left(A_{k}\right)∥}^{2}. \end{array}$$

In *ceres* [60], the optimum is computed in the least-squares sense with the Levenberg–Marquardt method:

(24) $$\begin{array}{c} \left[J{\left(A_{k}\right)}^{T}J\left(A_{k}\right)+\lambda_{k}I\right]\;\Delta A=-J{\left(A_{k}\right)}^{T}F\left(A_{k}\right), \end{array}$$

where $\lambda_{k}>0$ is a parameter and I is an identity matrix.

The algorithm is summarized in Algorithm 1.

**Algorithm 1**: Structure from Articulated Motion (SfAM)**Input:** initial normalized bone lengths $L_{b}$, measurement matrix $W\in R^{2T\times N}$ with 2D point tracks**Output:** poses $R\in R^{2T\times 3T}$ and 3D shapes $S\in R^{3T\times N}$**Initialize:**$S^{\left(0\right)}$ is initialized as in [11], $A^{\left(0\right)}=S^{\left(0\right)}$, β=1.5, $\mu^{\left(0\right)}=1$, ρ=0.25, τ=0.2**step 1:** recover R with IST method [57] (Section 3.2)**step 2 (optional):** smooth point trajectories in W [11]
**step 3: while not converged do**
    1: $A^{\left(t+1\right)}=\arg\min_{A}(\frac{\beta }{2}E_{BL}\left(A\right)+\frac{1}{2}\left|\right|S^{\left(t\right)}-A{\left|\right|}_{F}^{2})$    (optimize with Levenberg–Marquardt [55,56])    2: $g^{\left(t+1\right)}=R^{T}\left(RS^{\left(t\right)}-W\right)+\left(S^{\left(t\right)}-A^{\left(t+1\right)}\right)$    3: $Y^{\left(t+1\right)}=S^{\left(t\right)}-\tau g^{\left(t+1\right)}$    4: $S^{\left(t+1\right)}=S_{\tau \mu^{(}t)}\left(Y^{\left(t+1\right)}\right)$    5: $\mu^{\left(t+1\right)}=\mu^{\left(t\right)}\rho$
**end while**


## 4. Experiments and Results

We extensively evaluate our SfAM on several datasets including Human 3.6m [12], synthetic sequences of Akhter et al. [13] and NYU hand pose [14] dataset. Moreover, we demonstrate qualitative results on challenging community videos. In total, our SfAM is compared to over thirty state-of-the-art model-based and learning-based methods (see Table 1 and Table 2). We also implement SMSR of Ansari et al. [11], which is the most related approach to our SfAM and evaluate it on [12,14] as well as community videos. Moreover, we extend SMSR [11] with the local rigidity constraint of Rehan et al. [38] and include it into our comparison.

In Section 4.2.2, we evaluate the robustness of our approach to inaccuracies in 2D landmarks. The proposed SfAM recovers correct articulated structures given highly inaccurate initial bone lengths in Section 4.2.3. Finally, in Section 4.2.5, we highlight the numerous cases when our method performs better than state-of-the-art learning-based approaches in real-world scenes.

In all experiments, we use a sliding time window of 200 frames. For sequences shorter than 200 frames, we run our method on the whole sequence at once. All experiments are performed on a system with 32 GB RAM and twelve-core Intel Xeon CPU running at 3.6 GHz. Our framework is implemented in C++. Average processing time for a single frame from the Human 3.6m dataset [12] with given 2D annotations amounts to 140 ms.

### 4.1. Evaluation Methodology

We follow the established evaluation methodology in the area of NRSfM and rigidly align our 3D reconstructions to the ground truth. We report the reconstruction error $E_{3D}$ in mm between ground truth joint positions $\bar{S_{n}^{t}}$ and aligned 3D reconstructions $G(S_{n}^{t})$:

(25) $$\begin{array}{c} E_{3D}=\min_{G}\frac{1}{T}\frac{1}{N}\sum_{t=1}^{T}\sum_{n=1}^{N}\left|\right|\bar{S_{n}^{t}}-G\left(S_{n}^{t}\right){\left|\right|}_{2}, \end{array}$$

where n∈{1,…,N}, t∈{1,…,T}, *T* is the number of frames in the sequence and *N* is the number of joints of the articulated object. For some datasets, we report the normalized mean 3D error:

(26) $$\begin{array}{cc} \; & e_{3D}=\min_{G}\frac{1}{\sigma T}\frac{1}{N}\sum_{t=1}^{T}\sum_{n=1}^{N}\left|\right|\bar{S_{n}^{t}}-G\left(S_{n}^{t}\right){\left|\right|}_{2}^{2},\;\mathrm{with}\\ \; & \;\;\;\;\;\;\;\;\;\;\;\sigma =\min_{G}\frac{1}{3T}\sum_{t=1}^{T}\left(\sigma_{tx}+\sigma_{ty}+\sigma_{tz}\right), \end{array}$$

where $\sigma_{tx},\sigma_{ty}$ and $\sigma_{tz}$ denote normalized variances of reconstructions $G(S_{n}^{t})$ along the x,y,z-axes respectively.

### 4.2. Human Pose Estimation

#### 4.2.1. Human 3.6m Dataset

**Human 3.6m** [12] is currently the largest dataset for monocular 3D human pose sensing. It is widely used for evaluation of learning-based human pose estimation methods. Table 1 gives an overview of the quantitative results on the Human 3.6m [12]. We highlight approaches that are trained on Human 3.6m [12] with “*”. We follow three common evaluation protocols. In **Protocol #1**, we compare the methods on two subjects (S9 and S11). The original framerate 50 fps is reduced to 10 fps. The learning-based approaches marked with “*” use subjects S1, S5, S6, S7, S8 and all camera views for training. Testing is done for all cameras. For **Protocol #2**, only the frontal view (“camera3”) is used for evaluation. For **Protocol #3**, evaluation is done on every 64th frame of subject S11 for all cameras. The learning-based approaches marked with “*” use subjects S1, S5, S6, S7, S8 and S9 for training.

For all methods and under all evaluation protocols, we report the reconstruction error $E_{3D}$ after the rigid alignment of the recovered structures with ground truth. In our method, the bone lengths are initialized with the average values for all the subjects from the dataset.

As we see from Table 1, we show competitive accuracy to best performing learning-based approaches that are trained on Human 3.6m [12]. In Section 4.2.5, we demonstrate that our approach works better in real-world scenes which are different from this dataset.

**Figure 4:**
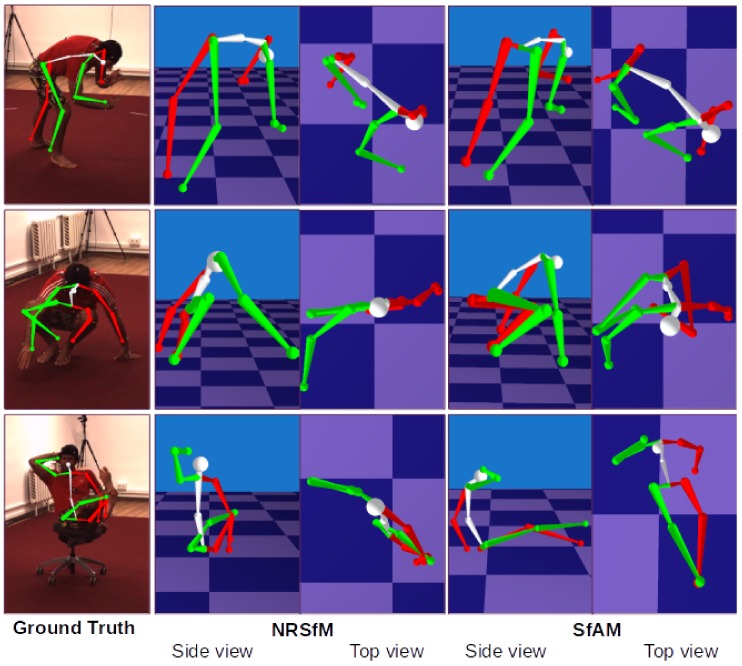
Comparison of our SfAM and NRSfM [11] on Human 3.6m [12]. NRSfM considers humans as general non-rigid objects and changes bone lengths from frame to frame.

**Figure A1:**
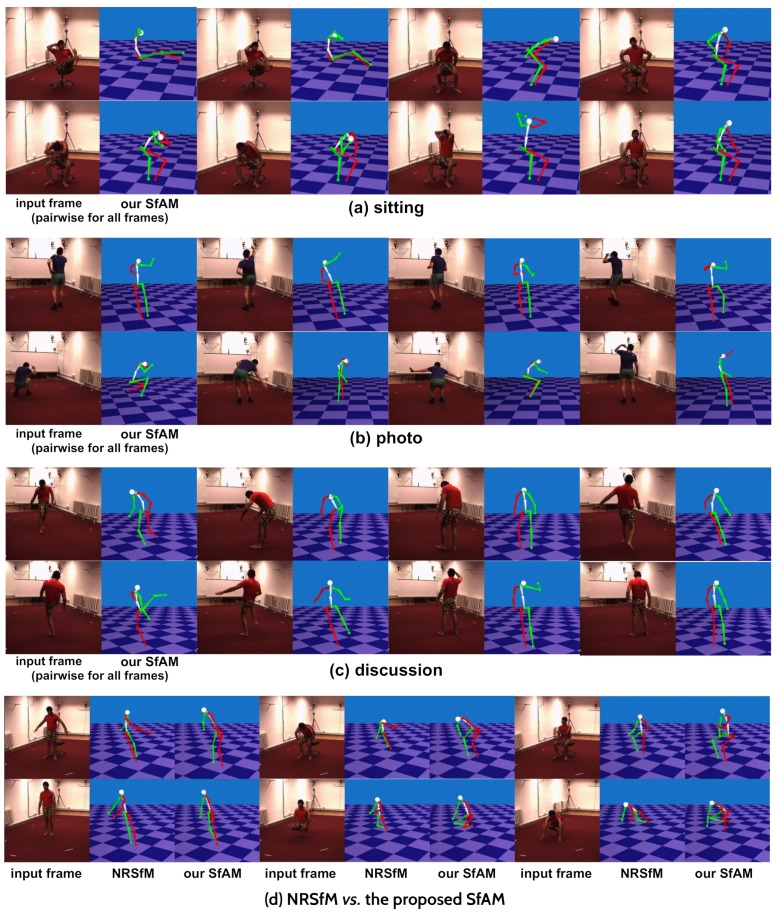
Additional visualizations of our results and reconstructions with NRSfM of Ansari et al. [11] on several sequences from [12]. (**a**)–(**c**): our results on *sitting*, *photo* and *discussion*. These sequences and poses are among the most challenging in the dataset. (**d**): comparison of our SfAM and NRSfM [11].

In Figure 4, we visualize several reconstructions of highly challenging scenes by SMSR [11] and the proposed SfAM. See Figure A1 for additional visualizations.

#### 4.2.2. Robustness to Inaccurate 2D Point Tracks

**Figure 5:**
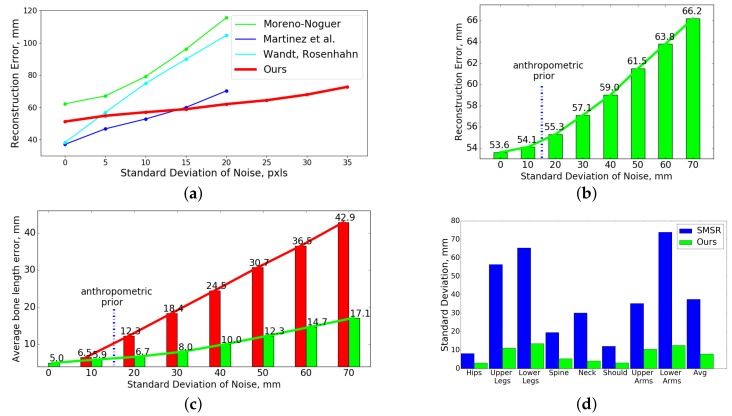
(**a**): the reconstruction error $e_{3D}$ under 2D noise; (**b**): $e_{3D}$ under incorrect bone lengths initializations; (**c**): average bone lengths error for the increasing levels of Gaussian noise before (red) and after (green) the optimization; (**d**): standard deviation of bone lengths for SMSR [11] and our SfAM.

We validate the robustness of our approach to inaccuracies in 2D landmarks on Human 3.6m [12]. We compare our SfAM to state-of-the-art learning-based methods [9,47,53] trained on ground truth 2D data. We add Gaussian noise with increasing values of the standard deviation to the 2D ground truth point tracks. The reconstruction error as the function of the standard deviation of the noise is plotted in Figure 5a. SfAM is more robust than the compared methods for moderate and high perturbations, and the error grows very slowly with the increasing noise level. In contrast to our SfAM, the errors of [9,47,53] grow very fast even with a low level of noise. Note that we evaluate our method on a higher level of noise than [9,47,53]. The average error of the currently best performing 2D detectors is between 10–15 pixels [79,80]. We see that, for 10–15 pixels, SfAM has comparable error to the most accurate learning-based approaches while not relying on training data and being generalizable for different object classes.

#### 4.2.3. Robustness to Incorrectly Initialized Bone Lengths and Real Bone Length Recovery

We study the accuracy of SfAM in recovering articulated structures given incorrectly initialized bone proportions (normalized bone lengths) on the subject S11 from Human 3.6m [12]. Starting from the ground truth initialization of bone lengths (obtained from the dataset), we change every bone length by adding different amounts of Gaussian noise with increasing standard deviations in the range [0;70] mm. This allows us to analyze the recovered bone lengths and the robustness of SfAM to noise in a controlled and well-defined setting. The results of the experiment are plotted in Figure 5b. If the structure is initialized with anthropometric priors from [81], the error increases by only 3%. Note that our error in bone length estimation is slightly affected by the increasing levels of noise. It is equal to 54 mm with ground truth initialization and grows just to 66 mm with σ=70 mm. Note that the anthropometric prior corresponds to σ≈15 mm.

Given incorrect initial bone lengths, SfAM recovers not only correct poses, but also accurate sequence-specific bone lengths. We calculate the average difference between ground truth bone lengths of subject S11 and the initial ones, provided to our method. We do the same for the recovered structures. The results are best viewed in Figure 5c. Thus, SfAM can be used for precise skeleton estimation.

We also calculate standard deviations of bone lengths of the reconstructed objects for SMSR [11] and SfAM. Figure 5d shows that the standard deviation of bone lengths is very high for SMSR [11], as it considers a human as a general non-rigid object and changes the bone lengths from frame to frame. SfAM reduces the average standard deviation by 514% leading to a more accurate pose reconstruction and structure recovery. In Figure 5d, “Upper Legs” and “Lower Legs” denote bones between the hip/knee and knee/ankle, respectively; “Upper Arms” and “Lower Arms” denote bones between shoulder/elbow and elbow/wrist, respectively.

#### 4.2.4. Synthetic NRSfM Datasets

**Synthetic sequences** of Akhter et al. [13] are commonly used for the evaluation of sparse NRSfM. We compare our approach with previous SfM methods on challenging synthetic sequences with a large variety of human motions *Drink*, *Pickup*, *Stretch*, and *Yoga* [20]. Some pairs of joints remain locally rigid in these sequences. We activate the articulated constraint for those points and evaluate our method. Table 2 shows the results of SfAM and previous SfM methods.

The errors $e_{3D}$ for other listed methods are taken from PPTA [78] and SMSR [11]. Only PPTA [78] outperforms SfAM on *Drink*, whereas CSF2 [23] achieves a comparable $e_{3D}$. SfAM achieves the most consistent performance among all compared algorithms.

#### 4.2.5. Real-World Videos

**Figure 6:**
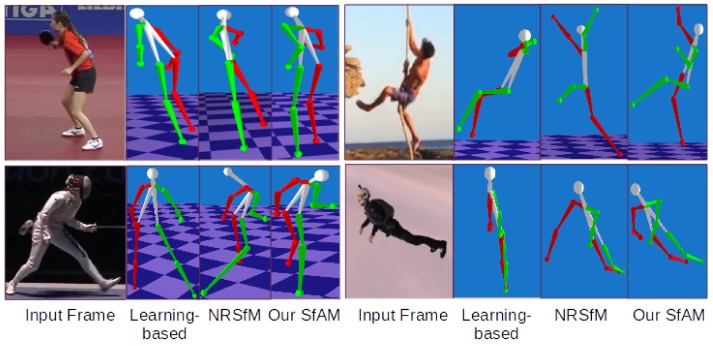
Comparison of our SfAM, NRSfM [11], and the learning-based method of Martinez et al. [9] on challenging real-world videos.

Our algorithm is capable of recovering human motion from challenging real-world videos. We compare our results with the state-of-the-art learning-based approach of Martinez et al. [9] and one of the best performing general-purpose NRSfM methods SMSR [11]. Since ground truth 2D annotations are not available, we use OpenPose [82] for 2D human body landmark extraction. Bone lengths are initialized with the values from *anthropometric data tables* [81]. As Figure 6 shows, [9] fails to correctly recover poses that are different from the training dataset [12]. SMSR [11] produces unrealistic human body structures. In contrast to [9,11], our method successfully recovers 3D human poses in real-world scenes.

### 4.3. Hand Pose Estimation

**Figure 7:**
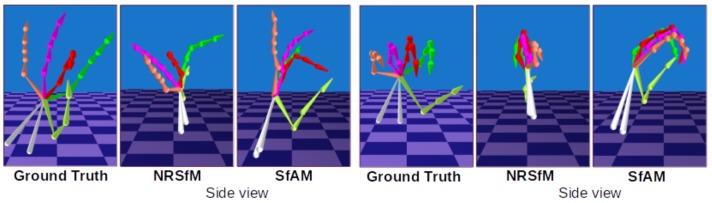
Comparison of our SfAM to NRSfM [11] on an NYU hand pose dataset [14].

We also evaluate SfAM on the NYU hand pose dataset [14], which provides 2D and 3D ground truth annotations for 8252 different hand poses. The hand model consists of 30 bones. Hand pose recovery is a challenging problem due to occlusion and many degrees of freedom. We compare the performance of our approach with SMSR [11] and its modification with local rigidity constraint from Rehan et al. [38]. Quantitatively, SfAM achieves $E_{3D}$ of 14.2 mm. In contrast, $E_{3D}$ of SMSR [11] is 22.2 mm, and SMSR with articulated body constraints [38] shows $E_{3D}$ of 19.4 mm. Hence, the inclusion of our articulated prior term to [11] achieves an error improvement of 56%. The qualitative results are shown in Figure 7. Similar to human bodies, SfAM achieves lower error due to keeping bone lengths constant between frames. When SMSR [11] fails to reconstruct the correct 3D pose, SfAM still outputs plausible results.

## 5. Conclusions

We present a new method for 3D articulated structure recovery from 2D landmarks. The proposed approach is general and not restricted to specific structures or motions. Integration of our soft articulated prior term into a general-purpose NRSfM approach and alternating optimization resulted in accurate and stable results.

In contrast to the vast majority of state-of-the-art approaches, SfAM does not require training data or known bone lengths. By ensuring consistency of bone lengths throughout the whole sequence, it optimizes sequence-specific bone proportions and recovers 3D structures. In extensive experiments, it proves its generalizability and shows accuracy close to state-of-the-art on public benchmarks. It also shows a remarkable improvement in accuracy compared to other model-based approaches. Moreover, our method outperforms learning-based approaches in complicated real-world videos. All in all, we show that high accuracy on benchmarks can be achieved without the need for training and parameter tuning for specific datasets.

In future work, we plan to apply SfAM to animal shape estimation and recovery of personalized human skeletons. We also believe it can boost the development of methods for human and hand pose estimation with semi-supervision.

## Figures and Tables

**Table 1:** The reconstruction error $E_{3D}$ of SfAM and previous methods on Human 3.6m dataset. “*” indicates learning-based methods which are trained on Human 3.6m [12]. We outperform all model-based approaches and reach very close to the tuned supervised learning techniques.

Method	P1	P2	P3
Zhou et al. [3] *	106.7	-	-
Akhter et al. [41]	-	181.1	-
Ramakrishna et al. [1]	-	157.3	-
Bogo et al. [61]	-	82.3	-
Kanazawa et al. [45] *	67.5	66.5	-
Moreno-Noguer [47] *	62.2	-	-
Yasin et al. [59]	-	-	110.2
Rogez et al. [62]	-	-	88.1
Chen, Ramanan [63] *	-	-	82.7
Nie et al. [64] *	-	-	79.5
Sun et al. [52] *	-	-	48.3
Omran et al. [65] *	59.9	-	-
Zhou et al. [66] *	54.7	-	-
Mehta et al. [8] *	54.6	-	-
Pavlakos et al. [67] *	51.9	-	-
Kinauer et al. [68] *	50.3	-	-
Tekin et al. [69] *	50.1	-	-
Rogez et al. [44] *	49.2	51.1	42.7
Habibie et al. [70] *	49.2	-	-
Martinez et al. [9] *	45.6	-	-
Zhao et al. [71] *	43.8	-	-
Pavlakos et al. [46] *	41.8	-	-
Arnab, Doersch et al. [72] *	41.6	-	-
Chen, Lin et al. [73] *	41.6	-	-
Sun et al. [74] *	40.6	-	-
Wandt, Rosenhahn [53] *	38.2	-	-
Pavllo et al. [75] *	36.5	-	-
Dabral et al. [58] *	36.3	-	-
SMSR [11]	106.6	105.2	102.9
SMSR [11]+[38]	145.2	124.0	139.9
Our SfAM	51.2	51.7	53.9

**Table 2:** The normalized mean 3D error $e_{3D}$ of previous NRSfM methods and our SfAM for synthetic sequences [20].

Method	Drink	PickUp	Stretch	Yoga
MP [76]	0.4604	0.4332	0.8549	0.8039
PTA [20]	0.0250	0.2369	0.1088	0.1625
CSF1 [77]	0.0223	0.2301	0.0710	0.1467
CSF2 [23]	0.0223	0.2277	**0.0684**	0.1465
BMM [10]	0.0266	**0.1731**	0.1034	**0.1150**
Lee [37]	0.8754	1.0689	0.9005	1.2276
PPTA [78]	**0.011**	0.235	0.084	0.158
SMSR [11]	0.0287	**0.2020**	0.0783	0.1493
SMSR [11]+[38]	0.4348	0.4965	0.3721	0.4471
Our SfAM	0.0226	**0.1921**	**0.0673**	**0.1242**

## Abbreviations

The following abbreviations are used in this manuscript:

SfAM	Structure from Articulated Motion
SfM	Structure from Motion
NRSfM	Non-Rigid Structure from Motion
FPC	Fixed-Point Continuation
SMSR	Scalable Monocular Surface Reconstruction
IST	Iterative Shrinkage-Thresholding

## References

[1] Ramakrishna V., Kanade T., Sheikh Y. Reconstructing 3D Human Pose from 2D Image Landmarks. Proceedings of the European Conference on Computer Vision (ECCV).

[2] Wandt B., Ackermann H., Rosenhahn B. (2016). 3D Reconstruction of Human Motion from Monocular Image Sequences. IEEE Trans. Pattern Anal. Mach. Intell. (TPAMI).

[3] Zhou X., Zhu M., Derpanis K., Daniilidis K. Sparseness Meets Deepness: 3D Human Pose Estimation from Monocular Video. Proceedings of the IEEE Conference on Computer Vision and Pattern Recognition (CVPR).

[4] Leonardos S., Zhou X., Daniilidis K. Articulated motion estimation from a monocular image sequence using spherical tangent bundles. Proceedings of the International Conference on Robotics and Automation (ICRA).

[5] Lee H.J., Chen Z. (1985). Determination of 3D human body postures from a single view. Comput. Vis. Graph. Image Process. (ICVGIP).

[6] Hossain M.R.I., Little J.J. Exploiting Temporal Information for 3D Human Pose Estimation. Proceedings of the European Conference on Computer Vision (ECCV).

[7] Zhou X., Huang Q., Sun X., Xue X., Wei Y. Towards 3D Human Pose Estimation in the Wild: A Weakly-Supervised Approach. Proceedings of the International Conference on Computer Vision (ICCV).

[8] Mehta D., Rhodin H., Casas D., Fua P., Sotnychenko O., Xu W., Theobalt C. Monocular 3D Human Pose Estimation in the Wild Using Improved CNN Supervision. Proceedings of the International Conference on 3D Vision (3DV).

[9] Martinez J., Hossain R., Romero J., Little J.J. A Simple However, Effective Baseline for 3d Human Pose Estimation. Proceedings of the International Conference on Computer Vision (ICCV).

[10] Dai Y., Li H., He M. (2014). A Simple Prior-Free Method for Non-rigid Structure-from-Motion Factorization. Int. J. Comput. Vis. (IJCV).

[11] Ansari M., Golyanik V., Stricker D. Scalable Dense Monocular Surface Reconstruction. Proceedings of the International Conference on 3D Vision (3DV).

[12] Ionescu C., Papava D., Olaru V., Sminchisescu C. (2014). Human3.6M: Large Scale Datasets and Predictive Methods for 3D Human Sensing in Natural Environments. IEEE Trans. Pattern Anal. Mach. Intell. (TPAMI).

[13] Akhter I., Sheikh Y., Khan S., Kanade T. (2011). Trajectory Space: A Dual Representation for Nonrigid Structure from Motion. IEEE Trans. Pattern Anal. Mach. Intell. (TPAMI).

[14] Tompson J., Stein M., Lecun Y., Perlin K. (2014). Real-Time Continuous Pose Recovery of Human Hands Using Convolutional Networks. ACM Trans. Graph. (ToG).

[15] Tomasi C., Kanade T. (1992). Shape and motion from image streams under orthography: A factorization method. Int. J. Comput. Vis. (IJCV).

[16] Bregler C., Hertzmann A., Biermann H. Recovering non-rigid 3D shape from image streams. Proceedings of the Computer Vision and Pattern Recognition (CVPR).

[17] Brand M. A direct method for 3D factorization of nonrigid motion observed in 2D. Proceedings of the Computer Vision and Pattern Recognition (CVPR).

[18] Xiao J., Chai J.X., Kanade T. A Closed-Form Solution to Non-rigid Shape and Motion Recovery. Proceedings of the European Conference on Computer Vision (ECCV).

[19] Bartoli A., Gay-Bellile V., Castellani U., Peyras J., Olsen S., Sayd P. Coarse-to-fine low-rank structure-from-motion. Proceedings of the Computer Vision and Pattern Recognition (CVPR).

[20] Akhter I., Sheikh Y., Khan S., Kanade T. Nonrigid Structure from Motion in Trajectory Space. Proceedings of the International Conference on Neural Information Processing Systems (NIPS).

[21] Hartley R., Vidal R. Perspective Nonrigid Shape and Motion Recovery. Proceedings of the European Conference on Computer Vision (ECCV).

[22] Akhter I., Sheikh Y., Khan S. In defense of orthonormality constraints for nonrigid structure from motion. Proceedings of the Computer Vision and Pattern Recognition (CVPR).

[23] Gotardo P.F.U., Martínez A.M. Non-rigid structure from motion with complementary rank-3 spaces. Proceedings of the Computer Vision and Pattern Recognition (CVPR).

[24] Zhu Y., Huang D., la Torre Frade F.D., Lucey S. Complex Non-Rigid Motion 3D Reconstruction by Union of Subspaces. Proceedings of the Computer Vision and Pattern Recognition (CVPR).

[25] Kumar S., Cherian A., Dai Y., Li H. Scalable Dense Non-Rigid Structure-From-Motion: A Grassmannian Perspective. Proceedings of the Computer Vision and Pattern Recognition (CVPR).

[26] Paladini M., Del Bue A., Xavier J., Agapito L., Stosić M., Dodig M. (2012). Optimal Metric Projections for Deformable and Articulated Structure-from-Motion. Int. J. Comput. Vis. (IJCV).

[27] Costeira J.P., Kanade T. (1998). A Multibody Factorization Method for Independently Moving Objects. Int. J. Comput. Vis. (IJCV).

[28] Tresadern P., Reid I. Articulated structure from motion by factorization. Proceedings of the Computer Vision and Pattern Recognition (CVPR).

[29] Yan J., Pollefeys M. (2008). A Factorization-Based Approach for Articulated Nonrigid Shape, Motion and Kinematic Chain Recovery From Video. IEEE Trans. Pattern Anal. Mach. Intell. (TPAMI).

[30] Fayad J., Russell C., Agapito L. Automated Articulated Structure and 3D Shape Recovery from Point Correspondences. Proceedings of the International Conference on Computer Vision (ICCV).

[31] Park H.S., Sheikh Y. 3D reconstruction of a smooth articulated trajectory from a monocular image sequence. Proceedings of the International Conference on Computer Vision (ICCV).

[32] Valmadre J., Zhu Y., Sridharan S., Lucey S. Efficient Articulated Trajectory Reconstruction Using Dynamic Programming and Filters. Proceedings of the European Conference on Computer Vision (ECCV).

[33] Kumar S., Dai Y., Li H. (2017). Spatio-temporal union of subspaces for multi-body non-rigid structure-from-motion. Pattern Recognit..

[34] Golyanik V., Jonas A., Stricker D. Consolidating Segmentwise Non-Rigid Structure from Motion. Proceedings of the International Conference on Machine Vision Applications (MVA).

[35] Taylor J., Jepson A.D., Kutulakos K.N. Non-rigid structure from locally-rigid motion. Proceedings of the Computer Vision and Pattern Recognition (CVPR).

[36] Fayad J., Agapito L., Del Bue A. Piecewise Quadratic Reconstruction of Non-Rigid Surfaces from Monocular Sequences. Proceedings of the European Conference on Computer Vision (ECCV).

[37] Lee M., Cho J., Oh S. Consensus of Non-rigid Reconstructions. Proceedings of the Computer Vision and Pattern Recognition (CVPR).

[38] Rehan A., Zaheer A., Akhter I., Saeed A., Mahmood B., Usmani M., Khan S. NRSfM using Local Rigidity. Proceedings of the Winter Conference on Applications of Computer Vision (WACV).

[39] Taylor C.J. Reconstruction of Articulated Objects from Point Correspondences in a Single Uncalibrated Image. Proceedings of the Computer Vision and Image Understanding (CVIU).

[40] Wei X.K., Chai J. Modeling 3D human poses from uncalibrated monocular images. Proceedings of the International Conference on Computer Vision (ICCV).

[41] Akhter I., Black M.J. Pose-conditioned joint angle limits for 3D human pose reconstruction. Proceedings of the Computer Vision and Pattern Recognition (CVPR).

[42] Wandt B., Ackermann H., Rosenhahn B. A Kinematic Chain Space for Monocular Motion Capture. Proceedings of the European Conference on Computer Vision Workshops (ECCVW).

[43] Xu W., Chatterjee A., Zollhöfer M., Rhodin H., Mehta D., Seidel H.P., Theobalt C. (2018). MonoPerfCap: Human Performance Capture From Monocular Video. ACM Trans. Graph. (ToG).

[44] Rogez G., Weinzaepfel P., Schmid C. (2019). LCR-Net++: Multi-person 2D and 3D Pose Detection in Natural Images. IEEE Trans. Pattern Anal. Mach. Intell. (TPAMI).

[45] Kanazawa A., Black M.J., Jacobs D.W., Malik J. End-to-End Recovery of Human Shape and Pose. Proceedings of the Computer Vision and Pattern Recognition (CVPR).

[46] Pavlakos G., Zhou X., Daniilidis K. Ordinal Depth Supervision for 3D Human Pose Estimation. Proceedings of the Computer Vision and Pattern Recognition (CVPR).

[47] Moreno-Noguer F. 3D Human Pose Estimation from a Single Image via Distance Matrix Regression. Proceedings of the Computer Vision and Pattern Recognition (CVPR).

[48] Malik J., Elhayek A., Stricker D. (2019). WHSP-Net: A Weakly-Supervised Approach for 3D Hand Shape and Pose Recovery from a Single Depth Image. Sensors.

[49] Malik J., Elhayek A., Stricker D. (2018). Structure-Aware 3D Hand Pose Regression from a Single Depth Image. International Conference on Virtual Reality and Augmented Reality.

[50] Malik J., Elhayek A., Nunnari F., Varanasi K., Tamaddon K., Heloir A., Stricker D. DeepHPS: End-to-end Estimation of 3D Hand Pose and Shape by Learning from Synthetic Depth. Proceedings of the International Conference on 3D Vision (3DV).

[51] Malik J., Elhayek A., Stricker D. Simultaneous Hand Pose and Skeleton Bone-Lengths Estimation from a Single Depth Image. Proceedings of the International Conference on 3D Vision (3DV).

[52] Sun X., Shang J., Liang S., Wei Y. Compositional Human Pose Regression. Proceedings of the International Conference on Computer Vision (ICCV).

[53] Wandt B., Rosenhahn B. RepNet: Weakly Supervised Training of an Adversarial Reprojection Network for 3D Human Pose Estimation. Proceedings of the Computer Vision and Pattern Recognition (CVPR).

[54] Ma S., Goldfarb D., Chen L. (2011). Fixed point and Bregman iterative methods for matrix rank minimization. Math. Program..

[55] Levenberg K. (1944). A method for the solution of certain nonlinear problems in least squares. Q. Appl. Math..

[56] Marquardt D.W. (1963). An algorithm for least-squares estimation of nonlinear parameters. J. Soc. Ind. Appl. Math..

[57] Beck A., Teboulle M. (2009). A fast iterative shrinkage-thresholding algorithm for linear inverse problems. SIAM J. Imaging Sci..

[58] Dabral R., Mundhada A., Kusupati U., Afaque S., Sharma A., Jain A. Learning 3D Human Pose from Structure and Motion. Proceedings of the European Conference on Computer Vision (ECCV).

[59] Yasin H., Iqbal U., Krüger B., Weber A., Gall J. (2015). 3D Pose Estimation from a Single Monocular Image. arXiv.

[60] Agarwal S., Mierle K. Ceres Solver. http://ceres-solver.org.

[61] Bogo F., Kanazawa A., Lassner C., Gehler P.V., Romero J., Black M.J. Keep It SMPL: Automatic Estimation of 3D Human Pose and Shape from a Single Image. Proceedings of the European Conference on Computer Vision (ECCV).

[62] Rogez G., Schmid C. MoCap-guided Data Augmentation for 3D Pose Estimation in the Wild. Proceedings of the International Conference on Neural Information Processing Systems (NIPS).

[63] Chen C., Ramanan D. 3D Human Pose Estimation = 2D Pose Estimation + Matching. Proceedings of the Computer Vision and Pattern Recognition (CVPR).

[64] Nie B.X., Wei P., Zhu S. Monocular 3D Human Pose Estimation by Predicting Depth on Joints. Proceedings of the International Conference on Computer Vision (ICCV).

[65] Omran M., Lassner C., Pons-Moll G., Gehler P.V., Schiele B. Neural Body Fitting: Unifying Deep Learning and Model Based Human Pose and Shape Estimation. Proceedings of the International Conference on 3D Vision (3DV).

[66] Zhou X., Zhu M., Pavlakos G., Leonardos S., Derpanis K.G., Daniilidis K. (2018). MonoCap: Monocular Human Motion Capture using a CNN Coupled with a Geometric Prior. IEEE Trans. Pattern Anal. Mach. Intell. (TPAMI).

[67] Pavlakos G., Zhou X., Derpanis K.G., Daniilidis K. Coarse-to-Fine Volumetric Prediction for Single-Image 3D Human Pose. Proceedings of the Computer Vision and Pattern Recognition (CVPR).

[68] Kinauer S., Güler R.A., Chandra S., Kokkinos I. Structured Output Prediction and Learning for Deep Monocular 3D Human Pose Estimation. Proceedings of the Energy Minimization Methods in Computer Vision and Pattern Recognition (EMMCVPR).

[69] Tekin B., Márquez-Neila P., Salzmann M., Fua P. Learning to Fuse 2D and 3D Image Cues for Monocular Body Pose Estimation. Proceedings of the International Conference on Computer Vision (ICCV).

[70] Habibie I., Xu W., Mehta D., Pons-Moll G., Theobalt C. In the Wild Human Pose Estimation Using Explicit 2D Features and Intermediate 3D Representations. Proceedings of the Computer Vision and Pattern Recognition (CVPR).

[71] Zhao L., Peng X., Tian Y., Kapadia M., Metaxas D.N. Semantic Graph Convolutional Networks for 3D Human Pose Regression. Proceedings of the Computer Vision and Pattern Recognition (CVPR).

[72] Arnab A., Doersch C., Zisserman A. Exploiting temporal context for 3D human pose estimation in the wild. Proceedings of the Computer Vision and Pattern Recognition (CVPR).

[73] Chen X., Lin K., Liu W., Qian C., Wang X., Lin L. Weakly-Supervised Discovery of Geometry-Aware Representation for 3D Human Pose Estimation. Proceedings of the Computer Vision and Pattern Recognition (CVPR).

[74] Sun X., Xiao B., Wei F., Liang S., Wei Y. Integral Human Pose Regression. Proceedings of the European Conference on Computer Vision (ECCV).

[75] Pavllo D., Feichtenhofer C., Grangier D., Auli M. 3D human pose estimation in video with temporal convolutions and semi-supervised training. Proceedings of the Computer Vision and Pattern Recognition (CVPR).

[76] Paladini M., Del Bue A., Stosic M., Dodig M., Xavier J.M.F., Agapito L. Factorization for non-rigid and articulated structure using metric projections. Proceedings of the Computer Vision and Pattern Recognition (CVPR).

[77] Gotardo P.F.U., Martinez A.M. Kernel non-rigid structure from motion. Proceedings of the International Conference on Computer Vision (ICCV).

[78] Agudo A., Moreno-Noguer F. (2018). A Scalable, Efficient, and Accurate Solution to Non-Rigid Structure from Motion. Comput. Vis. Image Underst. (CVIU).

[79] Wei S.E., Ramakrishna V., Kanade T., Sheikh Y. Convolutional Pose Machines. Proceedings of the Computer Vision and Pattern Recognition (CVPR).

[80] Newell A., Yang K., Deng J. Stacked Hourglass Networks for Human Pose Estimation. Proceedings of the European Conference on Computer Vision (ECCV).

[81] Gordon C.C., Churchill T., Clauser C.E., Bradtmiller B., McConville J.T. (1989). 1988 Anthropometric Survey of U.S. Army Personnel: Methods and Summary Statistics.

[82] Cao Z., Simon T., Wei S.E., Sheikh Y. Realtime Multi-Person 2D Pose Estimation using Part Affinity Fields. Proceedings of the Computer Vision and Pattern Recognition (CVPR).

